# Recurrence Rate of Trigeminal Neuralgia With the Use of Percutaneous Stereotactic Continuous Radiofrequency Ablation at 80°C for 90 Seconds: A Single-Center Study

**DOI:** 10.7759/cureus.21453

**Published:** 2022-01-20

**Authors:** Muhammad Hasan Wasim, Salman A Saleem, Sidra A Naqvi, Muhammad Nafees-ul Hasan, Naveed Ahmad Durrani, Muhammad Zubair

**Affiliations:** 1 Pain Management, Shifa International Hospital Islamabad, Islamabad, PAK

**Keywords:** trigeminal ganglion, stereotactic rhizotomy, recurrence rate, radiofrequency ablation (rfa), trigeminal nerve

## Abstract

Introduction

With recent development in the treatment of trigeminal neuralgia (TN), percutaneous stereotactic rhizotomy is being widely used as an interventional technique. The purpose of this study was to find the recurrence rate of TN in patients who were treated with stereotactic rhizotomy at 80°C for 90 seconds, in a tertiary care set up in a developing country.

Methodology

A retrospective cohort study was conducted at Shifa International Hospital, Islamabad, Pakistan from September 2016 to August 31, 2021. A total of 57 patients (19 males and 38 females) aged 27-90 years old, whose MRI of the brain had ruled out organic or structural pathologies, and who fulfilled the International Classification of Headache Disorders, 3rd edition for TN were recruited for the study. Of these patients, 51 underwent radiofrequency ablation (RFA) of the trigeminal ganglion (one or more branches of the trigeminal nerve (cranial nerve V (CN V)) in the operation theater. Patients having concomitant comorbid conditions like brain tumors, vascular pathologies, or coagulopathies, those who had previously undergone trigeminal ganglion neurolysis with either alcohol or phenol, who were lost to follow-up before the completion of the six months or had not visited back after the procedure, and those on oral anticoagulants and the ones declared high risk or American Society of Anesthesiologists (ASA) 3 and above for general anesthesia were excluded. Ethical approval was obtained and data were collected from the medical records department. The pain was recorded using the Numeric Rating Scale and recurrence was recorded from the follow-up visits of the patient over at least 12 months.

Results

Out of 51 patients, three patients who underwent RFA reported recurrence of the same problem for which they had initially reported to the pain clinic and were treated again with RFA. Five patients came back with the neuralgia of a different but contiguous branch of the same Gasserian ganglion opted for the RFA and were treated with no subsequent recurrences. The initial pain relief rate was 84.31%. At the end of the five-year study period, 16 patients reported variable degrees of sensory deficit, and two patients experienced non-debilitating unilateral reversible motor weakness of the jaw. One patient experienced keratitis due to unintentional loss of corneal reflex and subsequent ipsilateral loss of vision.

Conclusion

RFA is one of the leading treatment options for TN, with lower recurrence at higher temperatures of the radiofrequency electrode, at the cost of more significant sensory and motor deficits.

## Introduction

Trigeminal neuralgia (TN) is a severe chronic pain condition affecting the distribution of the cranial nerve V (CN V) in the head and facial region. TN has been defined by the International Headache Society as a disorder characterized by recurrent, unilateral, brief but very severe electric shock-like pains, abrupt in onset and termination, limited to the distribution of one or more divisions of the trigeminal nerve (CN V), and triggered by innocuous stimuli [1].

Previously known as tic douloureux or primary TN, it has been classified into three subtypes: classical, secondary, and idiopathic [1]. It is a form of neuropathic pain that can be precipitated secondary to another disorder or may occur primarily without any apparent cause. The diagnostic criteria given by the International Classification of Headache Disorders, 3rd edition (ICHD-3) include laterality (unilateral), radiation (not beyond CN V), distribution (CN V), intensity (severe), and three criteria, A, B, and C [1].

There have been multiple treatment modalities described in the texts including medical and surgical options to treat TN [2,3]. Since the individual course of the disease varies, most of the physicians adopt a step-by-step approach tailored to the individual needs of the patients starting preferentially from a medical therapeutic trial of anti-epileptic drugs, typically carbamazepine [4]. Other anticonvulsants that are prescribed include but are not limited to oxcarbazepine, gabapentin, and phenytoin [4]. Various surgical interventions are used to treat the condition, such as radiofrequency rhizotomies, balloon gangliolysis, stereotactic radiosurgery, and microvascular decompression to name a few [2,3].

During recent years in Pakistan, radiofrequency ablation (RFA) of the trigeminal ganglion has been employed across various centers with success, gradually replacing neurolytic techniques through a similar transforaminal approach [5,6]. Taha et al. used the "Cincinnati Paradigm" developed by Mayfield Clinic, based on the degree of response elicited at a specific voltage, to clinically achieve hypoalgesia and avoid sensory loss [7,8]. Raj et al. also described lesion parameters at 65°C, 70°C, and 75°C for 60 seconds, and more than two branches involving the fourth lesion have also been recommended [9].

The main objective is to find the recurrence rate of TN with the use of continuous RFA at 80°C for 90 seconds to target either single or multiple divisions of the ganglion at a tertiary care hospital, Shifa International Hospital, Pakistan.

## Materials and methods

A retrospective observational study was conducted at Shifa International Hospital, Islamabad, and patients who underwent RFA of the trigeminal ganglion (one or more divisions) from September 2016 to September 2020 were included in the study. The Institutional Review Board and Ethics Committee, Shifa International Hospital had issued approval (IRB#102-21). Patients aged between 27 and 90 years, whose MRI of the brain ruled out organic or structural pathologies and fulfilled ICHD criteria were recruited in the study, as indicated in Table 1.

**Table 1:** The International Classification of Headache Disorders diagnostic criteria for trigeminal neuralgia. CN V: cranial nerve V (trigeminal nerve); ICHD-3: International Classification of Headache Disorders, 3rd edition.

Recurrent paroxysms of unilateral facial pain in the distribution(s) of one or more divisions of the CN V, with no radiation beyond, and fulfilling criteria B and C.
A. Pain has all of the following characteristics: lasting from a fraction of a second to two minutes, severe intensity, electric shock-like, shooting, stabbing, or sharp in quality.
B. Precipitated by innocuous stimuli within the affected trigeminal distribution.
C. Not better accounted for by another ICHD-3 diagnosis.

Patients who had concomitant comorbid conditions like brain tumors, vascular pathologies, or coagulopathies were not recruited in the study. Moreover, those patients were also excluded who had previously undergone trigeminal ganglion neurolysis with alcohol, had lost to follow-up before the completion of the six months, had not visited back after the procedure, were on oral anticoagulants, or were declared high risk or American Society of Anesthesiologists (ASA) 3 and above for general anesthesia.

After approval from the hospital's ethical committee, records of the patients who underwent stereotactic continuous radiofrequency rhizotomy for TN at the operation theater of SIH, under the locally developed protocol, were requested from the medical records department from September 2016 to August 2021. We analyzed the data for recurrence rate, which was defined as patients complaining of similar symptoms within 12 months after the procedure requiring repeat intervention. The pain was the primary variable recorded from the available confidential records, quantified using the Numeric Rating Scale. Recurrence rate was recorded as patients coming back with similar complaints that they had initially presented with and treated for by the ablation, within 12 months after the procedure, requiring repeated procedures. The study was registered on clinicaltrials.gov (ID: NCT05101577).

## Results

There were a total of 57 patients enrolled in the study from 1st September 2016 to 31st August 2020 and followed until 31st August 2021. The patients diagnosed with TN had failed treatment with conventional medications like carbamazepine before they were enrolled for RFA because RFA is presumably reserved for patients who failed this treatment because of the associated risks. The patients diagnosed with TN in our study had an age range between 27 to 90 years (53.68 ± 14.52). Of 57 patients, six did not undergo RFA due to various reasons including financial and circumstantial restraints; one patient got better with medications alone and was subsequently lost to follow-up; one patient had a diagnostic block, and he did not opt for RFA. There were no recurrences in 43 of the 51 individuals who underwent RFA. Therefore, the initial pain relief rate was 84.31%. Pain scores were recorded using the Numeric Rating Scale.

**Figure 1:**
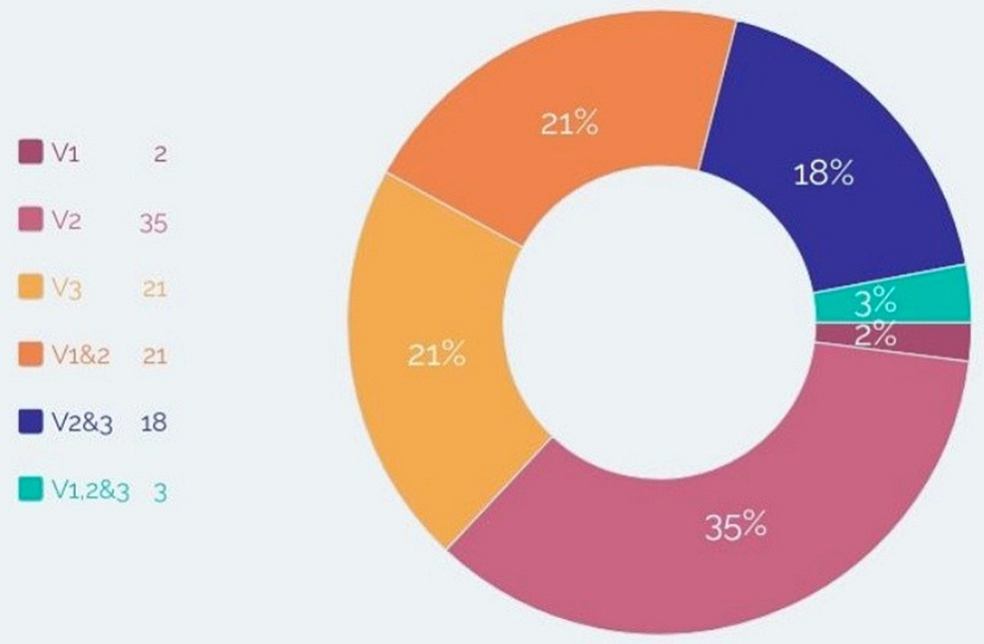
Distribution of trigeminal neuralgia symptoms by the regions involved. * Represented in the form of percentages.

Of the total 57 patients, there were 19 males (33%) and 38 females (66%). By 31st August 2021, one male and five female patients had reported recurrence after at least one intervention at the end of the study period of five years. A total of 25 patients (42.1%) had left-sided TN while 31 (57.9%) had presented with right-sided facial pains. A total of 33 patients had involvement of only division of the Gasserian ganglion with maxillary division (V2) being the most affected (21), followed by 11 patients having mandibular division (V3) and one patient reported pain along ophthalmic (V1) distribution. A total of 22 patients (39%) had neuralgia along two contiguous nerves, either V1 and V2 or V2 and V3. Only two patients had presented with TN along with the distribution of all three branches of the CN V (Figure 1).

Of the 51 patients who underwent RFA of the trigeminal ganglion, eight patients reported recurrence within 12 months of the intervention. Of those eight patients, three patients reported pain reappearing along with the same distribution for which they were initially treated. Five patients came back with pain along the different distribution for which rhizotomy had been done. Of the three patients coming back to us with the same problem within 12 months after we had performed stereotactic rhizotomy, two were admitted as daycare cases for repeat procedures, and one patient who did not consent to repeat procedure was given a trial of oxcarbazepine. Out of the two patients, one male and another female, the male patient reported complete pain relief and was eventually pain-free after a few months of the follow-up. The female patient had a recurrence for the third time after two years of the second intervention of the same division (right V2) and she also developed keratitis because of loss of corneal reflex (on the same side) due to inability to follow-up after the first intervention within four weeks. She was referred to an ophthalmologist for further management and had gone under regular follow-ups. We noted there were 16 patients (32%) who developed variable degrees of sensory deficits, including facial hypesthesia, the plastic-like feel of the affected side, which were reversed within 12 months in the majority of cases, except one female patient mentioned above who developed keratitis after the loss of corneal reflex. No patients were identified to have developed anesthesia dolorosa or complete masseter paralysis. No other ocular cranial nerves were affected in any of the patients. Two (4%) patients had partial motor deficits of V3, which were corrected with physiotherapy and rehabilitation. Our protocol for the stereotactic rhizotomy (higher temperature of the probe tip and higher duration) yielded lower recurrence rates for the TN with 6.19% as compared to previous studies and a re-intervention rate of 4% over a period of five years at the cost of significant sensory deficits (32%). At the end of the study period, an overall recurrence rate of 15.6% (eight patients) was recorded; three patients had same-region complaints, and five developed neuralgias in other divisions of CN V.

## Discussion

Treating TN using interventional procedures is a safe and effective treatment modality; however, it is limited by the anatomical difficulties faced by physicians. One of the areas most critical for identification and further intervention is the foramen ovale (FO). The FO is located in the middle cranial fossa in the greater wing of the sphenoid bone and transmits the third division of the CN V, the mandibular branch along with accessory meningeal artery emissary veins, otic ganglion, and sometimes lesser petrosal nerves and nervus spinosus [10]. It provides access to the operator for all three divisions of the trigeminal ganglion to be targeted by radiological guidance.

The majority of protocols and parameters for radiofrequency rhizotomies, neurotomies, and ablative procedures have been devised in the western centers of excellence with a specific set of environmental and social factors that come into play [9,11]. Taking into consideration the socio-economic statuses of the local populace and current health systems of Pakistan where the concept of insured health care is still scarce and the financial burden is more often than not borne by the patients, families, or caregivers, we improvised this protocol in an attempt to minimize the incidence of recurrence. Lower temperatures of the thermocouple tip for less time (65°C, 70°C, or 75°C for 60 seconds) resulted in a 20% recurrence rate of the trigeminal pain (including mild pain and that requiring no intervention), with the advantage of having minimal sensory loss of the orofacial region [7]. We assume that higher temperature and more time (80°C for 90 seconds) yield lower recurrence, but at the cost of higher (reversible) sensory deficits, translating into less repeat procedures and subsequently lower suffering and cost for the patients who suffer from the problem once dubbed as the “the suicide disease” [12].

**Figure 2:**
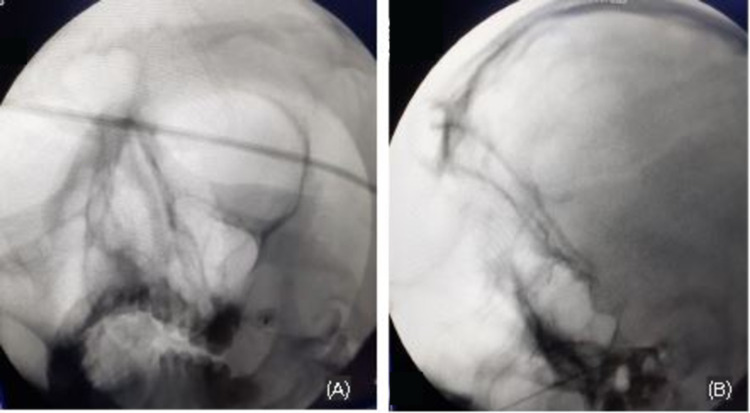
(A) Sub-mental view of cannulation through foramen ovale. (B) Lateral view of the electrode prior to radiofrequency ablation.

A cadaveric study undertaken by Reymond et al. showed that 4.5% of skulls have foramina divided into two or three compartments having radiological implications when accessing the target fluoroscopically or with CT guidance [13]. Another study on the Malay population showed the presence of accessory FO of about 0.1 cm diameter in 3.33% of the subjects (accessory FO) [14]. Elnashar et al. carried out a study on 174 adult human dry skulls, analyzing 348 foramina, and found out that there were six distinctive shapes and five anomalous variants of FO, making cannulation difficult in 8% of the subjects and calculated the risk of the inadvertent foramen lacerum cannulation to be 12% [11]. In our institution, we have relied on the radiological appearance under fluoroscopic guidance after marking the entry point in the safe zone of the face, to avoid the transition of the introducing cannula through the oral cavity. The ganglia were accessed without any obvious difficulty in all 51 patients during our study (see Figure 2A). Multiple approaches used to access the FO have been described by different authors, mostly having the same end-point, targeting the introducing cannula towards the junction of clivus and petrous part of the temporal bone in the axial or tunnel view. We follow a technique similar to that described by Aaron Cohen-Gadol, where the entry point is at 2.5 cm from the angle of the mouth (see Figure 2B) [15].

After ensuring adequate introducer needle placement, the sensory and motor stimuli were given to ascertain the position of the thermocouple tip at the exact point. Sensory and motor stimuli were given at 50 Hz and 2 Hz, respectively, to elicit paraesthesias and motor responses of the masseter muscle in all patients. After confirming the adequate target, a minimum of three lesions were given at 80°C for 90 seconds; both timing and temperature are more than conventionally described by Taha et al., Raj et al., and Awad and Mohamed [8,9,16].

In a prospective study over 15 years, 154 patients treated under Cincinnati Paradigm were followed up for recurrence after the stereotactic rhizotomy, and recurrence correlated directly with the degree of sensory deficit; 60% recurrence in patients with mild hypesthesia and 25% with dense sensory loss, and 20% with those who experienced analgesia [8]. In a recent study by Bharti et al., the authors measured the pain relief in patients whom they treated with three lesions at 70 degrees for 60 seconds each, for up to three months, reporting 67% pain relief [17]. Zheng et al. analyzed the data over 12 years, from 2005 to 2017, and found that in 1,481 patients from multiple centers undergoing radiofrequency thermocoagulation, the rate of recurrence-free interval significantly decreased over years. After one year, 85.3% of patients were symptoms free; 74.6% of patients were symptoms free at three years and 68.0% at five years, translating to approximately 32% recurrence of symptoms at the end of five years [18]. Comparing these data, we believe that the temperature of the thermoablation probe tip can be increased to 80°C and the time for the contact of a 5 mm active tip can be increased to 90 seconds for a single lesion to attain lower recurrence rates but the frequency of sensory deficit remains high.

Despite the fact that TN is uncommon and it is difficult to gather data on a sizable population, we have seen an influx of these patients and we have managed to treat a modest number using our institutional protocol for stereotactic rhizotomy. Owing to the disparity in health care and the burden of disease seen in patients, we chose a protocol that can effectively treat this condition as well as reduce the rate of recurrence albeit transient reversible sensory and motor deficits have been recorded in a small number of patients. We now advise the patients who undergo the intervention routinely about eye care and educate them about the importance of rehabilitative physiotherapy. This is to ensure that even if patients may be lost to follow-up following the intervention, they can remain reliably pain and complications-free. In our center, we are constantly updating our protocols as the results of follow-ups are coming in. We intend to follow these patients over the next five years and add more to the current pool because stereotactic rhizotomy of the trigeminal ganglion has been established as an effective modality for the treatment of TN over the years [19,20].

The main limitation was the number of patients whose records could be retrieved from the medical records department. The duration of the study period being five years meant that the first patients were followed for up to five years, but the last patients, treated in August 2020, were followed up for 12 months only. Therefore we made sure that all the patients were followed for at least 12 months. We intend to follow these patients further for a period of another five years. The dynamic and ever-evolving nature of the technological advancements in the treatment of such rare diseases warrants constant revision of the protocols and updates are made. We started collecting data from mid of 2020, during peak coronavirus disease 2019 (COVID-19) times. A formal request to retrieve the records was made in May 2020 for the patients treated from September 2016 onwards, and those patients who were under treatment from June 2020 onwards were followed prospectively. We started using the above-mentioned RFA protocol in September 2016; prior to that, chemical neurolysis with alcohol was being used to treat TN at our institution. We had observed significant complications despite having used the lowest possible volumes and a careful approach to the Meckel's cave; therefore, an RFA machine was requested in our setup.

## Conclusions

RFA has been established as a leading modality of treatment in TN. The results we achieved in our institution have been positive using our tailored protocol for our population, with significant but transient sensory deficits. We believe our protocol can be beneficial for patients especially in areas where healthcare access is limited and there is frequent loss of follow-ups due to social or financial reasons. Further large-scale studies can be helpful in establishing its efficacy and usefulness in other centers in Pakistan.
